# The Hippocampal Vulnerability to Herpes Simplex Virus Type I Infection: Relevance to Alzheimer’s Disease and Memory Impairment

**DOI:** 10.3389/fncel.2021.695738

**Published:** 2021-08-13

**Authors:** Shin Jie Yong, Min Hooi Yong, Seong Lin Teoh, Tomoko Soga, Ishwar Parhar, Jactty Chew, Wei Ling Lim

**Affiliations:** ^1^Department of Biological Sciences, School of Medical and Life Sciences, Sunway University, Petaling Jaya, Malaysia; ^2^Department of Psychology, School of Medical and Life Sciences, Sunway University, Petaling Jaya, Malaysia; ^3^Aging Health and Well-being Research Centre, School of Medical and Life Sciences, Sunway University, Petaling Jaya, Malaysia; ^4^Department of Anatomy, Universiti Kebangsaan Malaysia Medical Centre, Kuala Lumpur, Malaysia; ^5^Jeffrey Cheah School of Medicine and Health Sciences, Brain Research Institute Monash Sunway, Monash University Malaysia, Subang Jaya, Malaysia

**Keywords:** herpes simplex virus, hippocampus, neurotropism, Alzheimer’s disease, memory impairment, infectious etiology

## Abstract

Herpes simplex virus type 1 (HSV-1) as a possible infectious etiology in Alzheimer’s disease (AD) has been proposed since the 1980s. The accumulating research thus far continues to support the association and a possible causal role of HSV-1 in the development of AD. HSV-1 has been shown to induce neuropathological and behavioral changes of AD, such as amyloid-beta accumulation, tau hyperphosphorylation, as well as memory and learning impairments in experimental settings. However, a neuroanatomical standpoint of HSV-1 tropism in the brain has not been emphasized in detail. In this review, we propose that the hippocampal vulnerability to HSV-1 infection plays a part in the development of AD and amnestic mild cognitive impairment (aMCI). Henceforth, this review draws on human studies to bridge HSV-1 to hippocampal-related brain disorders, namely AD and aMCI/MCI. Next, experimental models and clinical observations supporting the neurotropism or predilection of HSV-1 to infect the hippocampus are examined. Following this, factors and mechanisms predisposing the hippocampus to HSV-1 infection are discussed. In brief, the hippocampus has high levels of viral cellular receptors, neural stem or progenitor cells (NSCs/NPCs), glucocorticoid receptors (GRs) and amyloid precursor protein (APP) that support HSV-1 infectivity, as well as inadequate antiviral immunity against HSV-1. Currently, the established diseases HSV-1 causes are mucocutaneous lesions and encephalitis; however, this review revises that HSV-1 may also induce and/or contribute to hippocampal-related brain disorders, especially AD and aMCI/MCI.

## Introduction

Alzheimer’s disease (AD) is the leading neurodegenerative disease, accounting for about 60-80% of dementia cases globally (Qiu et al., 2009; Alzheimer’s Association., 2021). AD may progress from a long period of subtle memory decline called amnestic mild cognitive impairment (aMCI; the most common type of MCI) (Petersen et al., 2001). While the etiology of AD is multifaceted, the hypothesis for an infectious cause in AD has emerged since the 1980s. Ball (1982) and Gannicliffe et al. (1986) first suggested that periodic reactivation of herpes simplex virus type-1 (HSV-1) from latency in neurons may facilitate the development of AD. In the following decades, the possible involvement of herpes viruses, such as HSV-1, HSV-2, cytomegalovirus (CMV), human herpesvirus types 6, 7, and 8 (HHV-6, -7, and -8), varicella-zoster virus (VZV) and Epstein-Barr virus (EBV), in AD and MCI have been investigated (Polk et al., 2002; Strandberg et al., 2003; Carbone et al., 2014; Barnes et al., 2015; Agostini et al., 2016b; Tsai et al., 2017; Tzeng et al., 2018). The collective evidence implicates HSV-1 as the most probable infectious agent contributing to AD and MCI, according to reviews and meta-analyses (Steel and Eslick, 2015; Itzhaki et al., 2016; Warren-Gash et al., 2019; Sait et al., 2021).

HSV-1 is an enveloped, linear double-stranded DNA virus that infects more than 60% of the population worldwide (Looker et al., 2015; Harfouche et al., 2019; Khadr et al., 2019). Productive infection of HSV-1, either from primary infection or latent reactivation, causes mucocutaneous lesion of the lips, cornea or genitals (Darougar et al., 1985; Scott et al., 1997; Ribes et al., 2001). HSV-1 also causes herpes simplex encephalitis (HSE), the most common type of infection-induced encephalitis (Granerod et al., 2010; George et al., 2014). In pregnant mothers with genital herpes, HSV-1 can cause congenital herpes in the infant upon vaginal delivery, resulting in mucocutaneous lesions and central nervous system (CNS) infection (Whitley et al., 1991; Whitley et al., 2007).

At the neuronal level, HSV-1 infection has been shown to induce tau hyperphosphorylation, amyloid-beta 40 and 42 (Aβ40/42) accumulation, oxidative stress, neuroinflammation and apoptotic dysregulation, all of which are implicated in the pathophysiology of neurodegenerative diseases such as AD. At the genetic level, gene products of the HSV-1 life cycle have been shown to interact with AD susceptibility genes, such as presenilin 1 and 2 (PSEN1 and PSEN2), apolipoprotein E allele 4 (ApoE4) and clusterin genes, to promote both viral infectivity and risk of AD. These molecular mechanisms of HSV-1-induced neuropathology in AD have been reviewed in Harris and Harris (2018) and Duarte et al. (2019). Consequently, at the behavioral level, HSV-1 infection has been found to induce memory and learning impairments reminiscent of AD (Beers et al., 1995; Armien et al., 2010; De Chiara et al., 2019).

While molecular mechanisms underpinning contributions of HSV-1 to AD have been reviewed extensively (Duarte et al., 2019; Marcocci et al., 2020), a neuroanatomical standpoint has not been considered in detail. Deciphering the HSV-1 infection pathway and tropism in the brain would advance the understanding of the potential neurological health outcomes of HSV-1 infection. This review, thus, examines which brain region is most affected by HSV-1. Literature to date suggests that it may be the hippocampus, given its cardinal role in learning and memory. The hippocampus and its neuronal connections to the entorhinal cortex, amygdala, olfactory bulb and hypothalamus comprise the limbic system (Vilensky et al., 1982). In the mammalian hippocampus, life-long neurogenesis has been shown to occur in the subgranular zone of the dentate gyrus (DG), where neural stem or progenitor cells (NSCs/NPCs) are localized (Altman and Das, 1965; Eriksson et al., 1998). The hippocampus is susceptible to various stressors, including chronic stress, aging and microbial infections. As a result, hippocampal functions such as learning and memory would be compromised. Hence, hippocampal dysfunction has been implicated in disorders that involve memory impairment as a symptom, such as depression, schizophrenia, dementia, aMCI/MCI and AD, as reviewed in Small et al. (2011) and Anand and Dhikav (2012).

This review first discusses the possible role of HSV-1 in the development of AD and aMCI/MCI in humans. Next, this review describes the mechanisms of HSV-1 infection in neurons and theoretical model of HSV-1 infection trajectory, focusing on its neurotropism or predilection to target the hippocampus based on cell culture, animal and human autopsy evidence. We suggest that the hippocampal vulnerability to HSV-1 infection may also present a pivotal factor in initiating or facilitating the development of aMCI/MCI and AD. Following this, factors and mechanisms affecting the hippocampal susceptibility to HSV-1 infection are discussed.

## Bridging HSV-1 to AD and Memory Impairment

HSE, either due to primary infection or viral reactivation, is known to cause long-term neurological sequelae despite immediate antiviral treatments (Riancho et al., 2013; Armangue et al., 2018). Damage to the temporal lobe and limbic system (especially the hippocampus), as well as impairments in memory and behavior (e.g., emotional instability and irritability), have been frequently observed among HSE survivors (Kapur et al., 1994; Caparros-Lefebvre et al., 1996; Dagsdottir et al., 2014; Harris et al., 2020). Degeneration of similar brain regions and consequent phenotypic abnormalities in HSE resemble that of AD. This observation has led to the hypothesis that repeated and periodic HSV-1 reactivation may contribute to AD development, especially in the aging population with declining immunocompetence (Ball, 1982; Esiri, 1982b; Gannicliffe et al., 1986). Therefore, this section will describe the relationship between HSV-1 and AD and memory impairment, which may also reflect prodromal AD, in humans.

### Alzheimer’s Disease

HSV-1 DNA has been detected within Aβ depositions in postmortem brain tissues of AD patients compared to non-AD controls (Mori et al., 2004). The same study also found HSV-1 antigens within cortical neurons, providing the first evidence of possible HSV-1 reactivation in the AD brain (Mori et al., 2004). A further study reported that most HSV-1 DNA was localized within Aβ plaques in the cortices of AD patients (Wozniak et al., 2009b). Furthermore, transcriptome analyses of brain specimens from cohorts of AD patients have revealed higher abundance of HSV-1 latency-associated transcripts (LATs; transcribed from HSV-1 DNA) than older adults without AD (Readhead et al., 2018). These results indicate that HSV-1 can infect the brain and is associated with AD neuropathology.

When compared to age-matched healthy controls, individuals with AD and aMCI exhibited increased levels of anti-HSV-1 IgG antibodies (Costa et al., 2017; Agostini et al., 2019; Pandey et al., 2019), which also correlated with increased cortical volumes (Mancuso et al., 2014a, b). Similarly, increased antibody levels and avidity index against HSV-1 were found to be elevated in aMCI patients that did not develop AD, compared to those who did. In the same study, HSV-1-specific antibody titers also correlated positively with hippocampal and amygdala volumes (Agostini et al., 2016a). Other studies have also found that aMCI patients displayed higher anti-HSV-1 IgG antibody levels and avidity index compared to that of both healthy controls and AD patients (Kobayashi et al., 2013; Costa et al., 2020). Taken together, these findings imply that robust antibody immunity against HSV-1 may prevent the brain atrophy progression of aMCI into AD, possibly via antibody neutralization of HSV-1 that protects the brain against HSV-1-induced neuropathology (Mancuso et al., 2014a; Agostini et al., 2016a; Costa et al., 2020).

When anti-HSV-1 immunity is inadequate to control HSV-1 infection, periodic HSV-1 reactivation and productive infection may occur. One nationwide retrospective cohort study in Taiwan reported that individuals diagnosed with recurrent HSV-1 infection had a 2.8-fold higher risk of developing AD than uninfected individuals. More importantly, antiherpetic medications reduced such risk by about 90% compared to placebo (Tzeng et al., 2018). In another nationwide retrospective cohort study involving participants with HSV-1 or VZV infections and uninfected matched controls in Sweden, antiherpetic treatment was associated with a 10% reduced risk of dementia. In untreated patients, the risk of dementia increased by 50% compared to uninfected controls (Lopatko Lindman et al., 2021). A four-national (i.e., Wales, Scotland, Denmark and Germany) retrospective cohort study found that persons with HSV infection who were not given anti-herpetic medication had 18% higher risk of dementia compared to uninfected controls, although this effect was present in the Germany cohort only (Schnier et al., 2021). In a smaller retrospective cohort study comprising HSV-1-seropositive older adults, antiherpetic prescription was associated with 70% lower risk of AD development compared to no prescription (Hemmingsson et al., 2021). Two aging prospective cohort studies have also found that the risk of AD was about twofold greater in those with IgM seropositivity for HSV-1 (Letenneur et al., 2008; Lovheim et al., 2015a). Taken together, these studies suggest that HSV-1 productive infection or reactivation may promote AD development, which may also be preventable with antiherpetic agents.

Interestingly, a longitudinal study reported that IgM seropositivity for HSV-1 was associated with memory decline, especially amongst carriers of ApoE4 (Lövheim et al., 2019). Likewise, in another prospective cohort study, ApoE4 carriers had a threefold increased risk of both AD and HSV-1 reactivation (i.e., as indicated by IgM seropositivity or elevated IgG levels) compared to ApoE4-negative individuals (Linard et al., 2020). Therefore, host genetic risk factors such as ApoE4 may modulate the interactions between HSV-1 and AD risk.

However, several studies found no significant differences in HSV-1 IgG seropositivity (Wozniak et al., 2005; Letenneur et al., 2008; Lovheim et al., 2015b) and HSV-1 DNA in the brain (Jamieson et al., 1991; Hemling et al., 2003; Pisa et al., 2017) between individuals with and without AD. This could be attributed to genetic factors that predispose HSV-1-infected individuals to AD. For instance, the presence of HSV-1 DNA or IgG seropositivity with ApoE4 gene has been shown to pose a greater risk factor in AD development than either one by itself (Lin et al., 1995, 1996; Itzhaki et al., 1997; Steel and Eslick, 2015; Lopatko Lindman et al., 2019). Another reason may be that HSV-1 IgG seropositivity and DNA only indicate a history of viral exposure, as HSV-1 may remain latent and non-infective. Other meta-analyses have found that HSV-1-specific IgM seropositivity and high IgG levels (i.e., indicating productive infection or reactivation) were associated with dementia and MCI, but not HSV-1 IgG seropositivity and DNA (Warren-Gash et al., 2019; Ou et al., 2020; Wu et al., 2020).

### Memory Impairment

As mounting evidence supports the link between HSV-1 infection and AD development, HSV-1 may also be associated with the prodromal stage of AD, aMCI. Patients with aMCI may show early signs of AD neuropathological attributes, such as hippocampal shrinkage, neurofibrillary tangles (NFT; aggregates of hyperphosphorylated tau) and Aβ40/42 accumulation, according to a systematic review (Stephan et al., 2012). Hippocampal neuroimaging has also been demonstrated to predict whether MCI patients would develop AD (Jack et al., 1999; Hu et al., 2016; Li et al., 2019). Thus, given that HSV-1 infection can induce hippocampal dysfunction, HSV-1 infection may also be associated with reduced memory function.

For instance, HSV-1 IgG seropositivity has been associated with an 18-fold increased odds of memory deficits in middle-aged adults (Dickerson et al., 2008). Associations between HSV-1 IgG seropositivity and impaired cognition were also reported in other groups, e.g., healthy soldiers (Fruchter et al., 2015), young individuals (Tarter et al., 2014; Vanyukov et al., 2018) and older adults (Zhao et al., 2020; Murphy et al., 2021). Therefore, the clinical biomarker of HSV-1 exposure (i.e., IgG seropositivity) is likely to be linked to impaired memory and other cognitive measures, while biomarkers of HSV-1 reactivation or productive infection (i.e., high IgG levels or IgM seropositivity) is linked to AD (Letenneur et al., 2008; Lovheim et al., 2015a; Warren-Gash et al., 2019). This indicates that HSV-1 reactivation or productive infection may promote more severe cognitive decline than mere HSV-1 exposure. In a population study comprising healthy adolescents, HSV-1 IgG seropositivity was associated with memory decline, whereas HSV-1 IgG levels correlated with both poor memory and executive functioning (Jonker et al., 2014).

Population studies have also reported that IgG levels specific for either HSV-1 or CMV independently predicted cognitive defect in the elderly (Strandberg et al., 2003), schizophrenics and their non-psychotic relatives (Shirts et al., 2008; Watson et al., 2013), and middle-aged adults (Tarter et al., 2014). However, some studies found that only CMV-specific (and not HSV-1-specific) IgG seropositivity or levels correlated with cognitive impairment in elderly populations (Aiello et al., 2006; Barnes et al., 2015; Nimgaonkar et al., 2016) and bipolar disorder patients (Tanaka et al., 2017). On the contrary, other studies showed that only HSV-1-specific IgG (and not CMV-specific) seropositivity or levels were associated with cognitive impairment in healthy adolescents (Jonker et al., 2014) and individuals with or without neuropsychiatric disorders (Dickerson et al., 2003; Yolken et al., 2011; Hamdani et al., 2017).

A putative explanation for these inconsistencies could be that both CMV and HSV-1 contribute to memory dysfunction. It was suggested that CMV, which is known to induce immune dysregulation, may exacerbate HSV-1-induced neurodegeneration, leading to AD (Stowe et al., 2012; Lovheim et al., 2018). This is based upon the finding that HSV-1-specific IgG levels increased with age only in CMV seropositive individuals (Stowe et al., 2012). CMV IgG seropositivity alone also did not elevate the risk of AD, but both CMV and HSV-1 seropositivity did (Lovheim et al., 2018), suggesting that CMV and HSV-1 interact to influence the risk of developing AD. For AD with severe memory impairment, HSV-1 likely plays a more predominant role in memory impairment than other herpesviruses, as suggested by meta-analyses (Steel and Eslick, 2015; Warren-Gash et al., 2019) and reviews (Itzhaki, 2014; Itzhaki and Klapper, 2014).

One key limitation of these studies is that HSV-1 seropositivity indicates the history of prior HSV-1 exposure. Seropositivity alone does not inform the status of HSV-1 infection; that is, active or latent infection, or viral replication in the central or peripheral nervous systems or peripheral epithelial cells (Dickerson et al., 2008; Murphy et al., 2021). Therefore, the possible link of causality or pathophysiological pathways between HSV-1 seropositivity and memory impairment remains unclear (Vanyukov et al., 2018). Besides, the majority of the global adult population (i.e., > 60%) is seropositive for HSV-1 (Looker et al., 2015; Harfouche et al., 2019; Khadr et al., 2019), whereas only relatively few develop memory impairment (i.e., 10–20% of adults over 50 years) (Overton et al., 2019; Pais et al., 2020; Lu et al., 2021). This suggests that HSV-1 seropositivity may only play a subtle role in the development of memory impairment in certain cases.

## HSV-1 in the Nervous System

HSV-1 has four notable structures: the glycoproteins-embedded membrane, tegument layer, capsid and double-stranded DNA genome (Grunewald et al., 2003). Although HSV-1 is considered a human pathogen, it can infect other animals in experimental settings, as reviewed in Karasneh and Shukla (2011). The primary target cells of HSV-1 are epithelial and neuronal cells, where HSV-1 can alternate between lytic and latent life cycle phases, as reviewed in Kukhanova et al. (2014). This section will detail the mechanisms of HSV-1-neuronal interactions, followed by the theoretical model of HSV-1 infection trajectory in the nervous system.

### HSV-1 Infection and Latency

First, HSV-1 attachment to cells depends on the binding of envelop glycoprotein B (gB) to surface heparan sulfate proteoglycans (HSPGs), with an optional gC to augment the interaction (WuDunn and Spear, 1989; Herold et al., 1991, 1994). Alternatively, gB can bind to myelin-associated glycoprotein (MAG) (Suenaga et al., 2009) and non-muscle myosin heavy chain IIA and IIB (NHMC-IIA and -IIB) to initiate viral entry (Arii et al., 2010, 2015). The subsequent binding of gD to 3-O-sulfated heparan sulfate, herpesvirus entry mediator (HVEM) and/or nectin-1 (Montgomery et al., 1996; Whitbeck et al., 1997; Geraghty et al., 1998; Shukla et al., 1999) catalyzes viral fusion and entry via activation of gH/gL (Atanasiu et al., 2010).

Following HSV-1 entry, the DNA-containing nucleocapsid and tegument proteins are released into the cell cytoplasm. One of the tegument proteins may proceed to turn off protein translation in the cell (Kwong and Frenkel, 1987). The nucleocapsid moves and docks on the nuclear pore, which then disassembles to release the DNA into the cell nucleus (Sodeik et al., 1997; Dohner et al., 2002). This initiates a cascade of HSV-1 gene expression, in the order of α-genes, β-genes and γ-genes, where viral structural proteins and DNA are synthesized and re-assembled (Honess and Roizman, 1974). The assembled viral particles may bud into the inner nuclear membrane and undergo primary envelopment for nuclear egress. HSV-1 particles may subsequently undergo de-envelopment at the outer nuclear membrane to allow for further viral assembly in the cytoplasm, and complete the secondary envelopment at Golgi vesicles before the mature virions egress out of the host cell (Hutchinson and Johnson, 1995; Granzow et al., 2001), as reviewed in Mettenleiter (2002).

After this lytic phase in peripheral epithelial cells, HSV-1 can proceed to the latent phase in neurons, typically in the trigeminal ganglion (Takasu et al., 1992; Theil et al., 2001; Hufner et al., 2009). The latent phase of HSV-1 starts similarly to the lytic phase, until HSV-1 reaches the nucleopore by retrograde axonal transport (Antinone and Smith, 2010). In the latent phase, HSV-1 lytic gene expression in the nucleus is silenced, and no infectious viral progeny is produced. Instead, HSV-1 DNA undergoes histone modifications with nucleosomes of the chromatin in cell nucleus (Deshmane and Fraser, 1989; Kubat et al., 2004; Wang et al., 2005). During latency, HSV-1 gene expression is limited to LATs and a few microRNAs, which may inhibit (i) lytic gene transcription to maintain latency and (ii) apoptosis to promote survival of infected neurons (Perng et al., 2000; Wang et al., 2005; Umbach et al., 2008).

HSV-1 can transition from latent to lytic phase under conditions of cellular stress, such as chemotherapy, hyperthermia, ultraviolet radiation, fever, immunosuppression and psychological stress, as reviewed in Suzich and Cliffe (2018) and Yan et al. (2020). During HSV-1 reactivation, the HSV-1 DNA-associated chromatin relaxes and transcription of the viral regulatory VP16 protein begins, which consequently activates a cascade of viral lytic gene expression that lead to the production of infectious viral progeny (Thompson et al., 2009; Kim et al., 2012; Sawtell and Thompson, 2016). Newly manufactured HSV-1 then travels by anterograde transport from the cell body to axon termini to infect neighboring epithelial cells or neurons (Snyder et al., 2008; Miranda-Saksena et al., 2009). HSV-1 reactivation can lead to the appearance of disease (e.g., cold sores and HSE), asymptomatic viral replication or spread into the CNS, as reviewed in Bearer (2012) and Marcocci et al. (2020).

Neurons with actively replicating HSV-1 via reactivation or primary infection begins to undergo various mechanisms that lead to pathological changes, as reviewed in Harris and Harris (2018) and Duarte et al. (2019). Neuronal culture studies have demonstrated that HSV-1 induced tau hyperphosphorylation by upregulating several enzymes such as caspase-3, protein kinase A and glycogen synthase kinase 3β (Wozniak et al., 2009a; Lerchundi et al., 2011; Alvarez et al., 2012). HSV-1-infected neurons have also been shown to exhibit impaired autophagy and amyloid precursor protein (APP) processing, resulting in increased Aβ40/42 accumulation (De Chiara et al., 2010; Santana et al., 2012; Piacentini et al., 2015). These HSV-1-induced AD-related neuropathology can be inhibited with antiviral treatment targeting HSV-1 *in vitro* (Wozniak et al., 2011, 2013, 2005). These studies indicate that HSV-1 spread into the brain may facilitate the development of AD-related neuropathology. Animal models further provided support wherein HSV-1 infection or reactivation have been shown to induce AD neuropathology in the brain, which was also associated with learning and memory impairments (Martin et al., 2014; De Chiara et al., 2019).

### HSV-1 Infection Trajectory: Emphasis on Hippocampal Tropism

In the brain, HSV-1 invasion has been shown to target the olfactory system and hippocampus, followed by the higher cortical areas in animal studies (Table 1). According to Braak’s staging scheme in human AD samples, the hippocampus–entorhinal circuitry within the temporal lobe deteriorates the earliest, followed by higher cortical areas (Braak and Braak, 1991). Increasing evidence has also suggested that dysfunction of the olfactory system may indicate prodromal AD in humans, as Murphy (2019) reviewed. With this anatomical resemblance, Fewster et al. (1991) and Ball et al. (2013) have previously suggested that HSV-1 might induce the neuron-to-neuron tauopathy and Aβ spread in AD as HSV-1 propagates along its infection pathways.

**TABLE 1:** Animal infection models assessing the neuronal invasion and spread of HSV-1.

Model of infection	Site of viral dissemination	Site of latency	Neurological and behavioral findings	References

Lip infection				
Tooth pulp inoculation into BALB/C mice (young; 5–6 weeks)	• Hippocampus • EC • TG • Amygdala • Brainstem • Insular cortex • Olfactory cortex • Cingulate cortex • Temporal cortex	N/A	N/A	Barnett et al., 1994
Infection by lip abrasion into C57BL/6 mice (newborn; PND 0–1)	• Hippocampus	• TG	Accumulation of Aβ40/42 peptides and reduced neurogenesis in the hippocampus.	Li Puma et al., 2019
Infection by lip abrasion into BALB/c and 3xTg-AD mice (young; 6–8 weeks)	• Hippocampus • Neocortex • Cerebellum	• TG	Upregulated neuroinflammatory (astrogliosis, IL-1β and IL-6) and neurodegenerative (Aβ40/42 and hyperphosphorylated tau) markers in neocortex and hippocampus. Mice exhibited learning and memory deficits.	De Chiara et al., 2019
			Upregulated oxidative stress markers (HNE, HNE-modified proteins, protein carbonyls and 3-nitrotyrosine) in cortex.	Protto et al., 2020

**Intranasal infection**				

Intranasal inoculation into BALB/C mice (young; 3–4 weeks)	• Hippocampus • OB • Amygdala • Hypothalamus • Brainstem	N/A	N/A	Anderson and Field, 1983
Intranasal inoculation into BALB/C mice (young; 6 weeks)	• Hippocampus • OB • Trigeminal root entry • Brainstem • Amygdala • Thalamus • Hypothalamus • Temporal lobe • Cingulate cortex	N/A	Neuronal degeneration and acute inflammation in infected areas, especially the trigeminal system.	Tomlinson and Esiri, 1983
Intranasal inoculation into BALB/C mice (young; 6–8 weeks)	• Hippocampus • EC • OB • Trigeminal nerve • Brainstem	N/A	N/A	Webb et al., 1989
Intranasal inoculation into New Zealand White rabbits (adult)	• EC • TG • OB • Olfactory cortex	• EC • TG • Olfactory cortex	Acute inflammation in olfactory structures, including the EC.	Stroop et al., 1990
Intranasal inoculation into Lewis rats (adult)	• Hippocampus • EC • Amygdala • TG • OB	• Hippocampus • EC • OB	Inflammatory and haemorrhagic lesions in the TG, OB, amygdala, EC, spinal trigeminal nuclei and hippocampus.	Beers et al., 1993
Viral injection into OB of Sprague-Dawley rats (adult)	• EC • Olfactory nucleus • Piriform cortex	N/A	Mice with bilateral damage to olfactory cortex exhibited impaired learning and memory.	McLean et al., 1993
Intranasal inoculation into Lewis rats, followed by a recovery period from HSE (adult)	• Hippocampus • EC	• Hippocampus • EC	Impairments in spatial memory and learning. Brain tissues remain histologically normal.	Beers et al., 1995
Intranasal inoculation into SJL/NBOM mice (adult)	• TG • Olfactory epithelium	N/A	Cytopathic effects found predominantly in the hippocampus, temporal and frontobasal lobes, thalamus, pons and mesencephalon.	Meyding-Lamade et al., 1998, 1999
Intranasal inoculation into Albino Swiss CD-1 mice (young; 6–10 weeks)	• Hippocampus • EC • OB • Amygdala • Frontal lobe • Temporal lobe	N/A	HSV-1 replicated in the brain without producing neurological or behavioral anomalies.	Boggian et al., 2000
Intranasal inoculation into BALB/C mice (young; 4–6 weeks)	• Temporal cortex	N/A	Aβ40/42 deposition in the temporal cortex.	Wozniak et al., 2007
Intranasal inoculation into Wistar Hannover GALAS rats (young; PND 14)	• Hippocampus • OB • TG • Cerebral cortex Medulla	N/A	Most viral antigens were localized in the DG subfield of the hippocampus. Severe neuronal loss and tissue damage in infected areas.	Ando et al., 2008
Intranasal inoculation into BALB/c mice to induce encephalitis (young; 8–10 weeks)	• Hippocampus • Trigeminal nerve • OB • Thalamus • Hypothalamus • Temporal cortex • Piriform cortex	N/A	Glial cells necrosis and myelin degeneration within the hippocampus and lateral tegmental nucleus. Neuronal loss in the hippocampus (most profound), EC, amygdala and temporal cortex. Lymphocytic infiltration in the hippocampus and temporal cortex. Mice exhibited severe learning deficits.	Armien et al., 2010
Intranasal inoculation into Sprague–Dawley rats (adult)	• Hippocampus • TG • OB • Brainstem	N/A	N/A	Jennische et al., 2015

**Corneal/Ocular infection**				

Corneal inoculation into BALB/C mice (young; 4-6 weeks)	• TG • Pons (entry of trigeminal nerve)	• TG • Pons • Cerebellum • EC • Hippocampus	N/A	Deatly et al., 1988
Corneal inoculation into Californian rabbits (young; 6 weeks)	• TG • Pons (entry of trigeminal nerve)	• TG • Pons	Profound neuroinflammation in the hippocampus and EC.	Paivarinta et al., 1994
Ocular infection into BALB/c mice (young; 10 weeks)	• Hippocampus • OB • Brainstem • Cerebellum • Frontal lobe	• Hippocampus • OB Brainstem Cerebellum Frontal lobe	N/A	Chen et al., 2006
Ocular infection into tree shrews (*Tupaia belangeri chinensis*; young; 6-months)	• Hippocampus • OB • Brainstem • Thalamus • Cerebral cortex	• OB • Brainstem	N/A	Li et al., 2016
Corneal infection into C57BL/6J mice (young; 10 weeks)	• Hippocampus • SVZ • NPCs • Midbrain • Frontal lobe	• TG • Hippocampus • SVZ • Midbrain • Frontal lobe	Chronic inflammation in the hippocampus, SVZ and midbrain.	Menendez et al., 2016

**Brain infection**				

Intracerebral inoculation into BALB/C mice (young; 3–4 weeks)	• Hippocampus • Hypothalamus • Cerebral cortex	N/A	N/A	Anderson and Field, 1983
HSV-1 vector propagation in an *ex vivo* system of brains of BALB/c mice and SABRA rats (newborn; PND 1–2 and young; 4 weeks)	• Hippocampus • NPCs • Ependymal cells • Ventricles • Cortical areas	N/A	N/A	Braun et al., 2006
Stereotactic injection of HSV-1 into the hippocampus of transgenic AD mice (5XFAD) (young; 5–6 weeks)	• Cortex	N/A	Accumulation of Aβ42 peptides in the brain, which inhibited HSV-1 infectivity and protected mice from acute viral encephalitis.	Eimer et al., 2018
Intracranial infection into transgenic 5xFAD mice (young; 3-months)	• Hippocampus • Cortex	N/A	Accumulation of Aβ42 peptides.	Ezzat et al., 2019
Intracranial infection into C57BL/6 mice (age and weight N/A)	• Hippocampus	N/A	Neuronal loss, upregulated inflammatory markers (TNF, IL1-β, IL-6 and IFNα/IFNβ) and suppressed anti-inflammatory (IL-10, SOCS2 and SOCS3) signals in the hippocampus.	Toscano et al., 2020

**Peripheral infection**				

Intravenous and sciatic nerve inoculation into BALB/C mice (young; 3–4 weeks)	• Brainstem • Hypothalamus	N/A	N/A	Anderson and Field, 1983
Intraperitoneally inoculation into female C57BL/6 mice (young; 14 weeks)	• Hippocampus • Ventricles • Midbrain • Cerebellum • Cortex	• TG • Hippocampus	N/A	Burgos et al., 2006

*Aβ, amyloid-beta; EC, entorhinal cortex; HNE, 4-hydroxynonenal; IL, interleukin; NPCs, neural progenitor cells; IFN, interferon; OB, olfactory bulb; PND, postnatal day; SOCS, suppressor of cytokine signaling; SVZ; subventricular zone; TG, trigeminal ganglion; TNF, tumor necrosis factor.*

**FIGURE 1:**
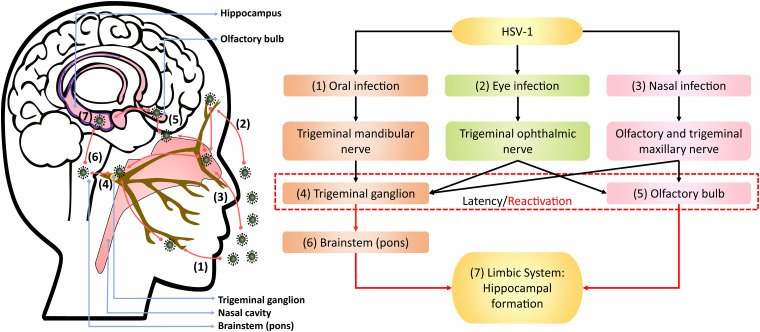
Theoretical model of HSV-1 infection trajectory within the nervous system. Following viral replication in the (1) oral, (2) corneal, or (3) olfactory epithelial cells, HSV-1 can exploit the neuronal retrograde machinery to reach the (4) trigeminal ganglion and (5) olfactory bulb, which are also sites for HSV-1 latency and stress-induced reactivation. Reactivated HSV-1 relies on anterograde transport to infiltrate the brain. Therein, HSV-1 travels from the trigeminal ganglion to the (6) pons, a part of brainstem innervated by the trigeminal nerve, and then to the (7) limbic system that inhabits the hippocampal-entorhinal circuitry. Alternatively, HSV-1 may directly infect the hippocampal-entorhinal circuity via the olfactory bulb, which is part of the limbic system.

Upon oral infection in animal models, HSV-1 can infect the mandibular trigeminal nerve to establish latency at the trigeminal ganglion (Barnett et al., 1994; Lewandowski et al., 2002; De Chiara et al., 2019). Alternatively, the nasal cavity can be an infection site wherein HSV-1 can travel along the olfactory and trigeminal maxillary nerves and become latent in the olfactory bulb and trigeminal ganglion of animals (Stroop et al., 1990; Beers et al., 1993; Jennische et al., 2015). Autopsy studies have also detected HSV-1 DNA, including LATs, in the trigeminal ganglion, trigeminal nerves and olfactory bulb of deceased humans (Liedtke et al., 1993; Theil et al., 2001; Hufner et al., 2009). In animal studies, HSV-1 could also infect the eye, propagating along the corneal subbasal nerve plexus innervated by trigeminal ophthalmic nerve, to initiate latency in the olfactory bulb and trigeminal ganglion (He et al., 2017; Menendez and Carr, 2017; Figure 1). Although congenital herpes is usually contracted upon vaginal delivery, *in utero* infection can occur in 5% of human infant cases (Hutto et al., 1987; Marquez et al., 2011). In such instances, using the murine model, genital HSV-1 likely enters the bloodstream and crosses the placenta into the fetal nervous system following the trigeminal infection route (Burgos et al., 2006).

Following reactivation from the trigeminal ganglion, HSV-1 may infiltrate the pons innervated by trigeminal nerves and then travel along the brainstem to the limbic system, as demonstrated in animal models (Tomlinson and Esiri, 1983; Webb et al., 1989; Barnett et al., 1994; Paivarinta et al., 1994). The olfactory bulb constitutes part of the limbic system and has direct projections to the hippocampus. Thus, reactivation from the olfactory bulb may provide HSV-1 and other neurotropic viruses direct access to the hippocampus, as proposed by Mori et al. (2005) and Duarte et al. (2019).

The theoretical model depicting neuronal pathways of HSV-1 infection in the brain (Figure 1) is also consistent with autopsy examinations of HSV-1 antigen distribution amongst HSE patients. Specifically, HSV-1 antigens were localized mostly in the hippocampus with the highest number of cases and viral abundance found, as well as in the temporal lobe, olfactory bulb and amygdala (Dinn, 1979; Twomey et al., 1979; Esiri, 1982a, b). Notably, postmortem analysis of AD victims detected HSV-1 DNA more frequently in the hippocampus and temporal cortex compared to other brain areas (Jamieson et al., 1991, 1992). Damasio and Van Hoesen (1985) also hypothesized that HSV-1 travels to the limbic system via the trigeminal nerve, wherein HSV-1 may exhibit higher affinity for the hippocampus, and subsequently spread to cortices during HSE.

Most animal models investigating HSV-1 neurotropism following reactivation, primary infection, or both have supported the predilection of HSV-1 to infect the hippocampus (Table 1). Some studies induced stress in animal models to reactivate HSV-1 and showed that the consequent viral replication was particularly prominent in the hippocampus (Burgos et al., 2006; De Chiara et al., 2019). Further supporting evidence can be derived from animal findings that demonstrated impairment in hippocampus-dependent memory and learning tasks following HSV-1 infection (McLean et al., 1993; Beers et al., 1995; Armien et al., 2010; De Chiara et al., 2019).

AD-associated neuropathology induced by HSV-1 can be observed in the hippocampus. For instance, multiple reactivations of HSV-1 caused memory deficits that were correlated with increased Aβ accumulation, tau phosphorylation and neuroinflammation in the neocortex and hippocampal DG of mice (De Chiara et al., 2019). It was demonstrated that HSV-1 could form a protein corona layer that served as catalytic surfaces for Aβ accumulation in the hippocampus and cortex of mice (Ezzat et al., 2019). Neurodegeneration and lymphocytic infiltration were also observed in the hippocampus, entorhinal cortex, amygdala and temporal cortex in HSV-1-infected mice (Ando et al., 2008; Armien et al., 2010; Toscano et al., 2020). Moreover, HSV-1 has been shown to inhibit the proliferation and differentiation of hippocampal NSCs (Li Puma et al., 2019). In mature hippocampal neurons, acute HSV-1 infection has been shown to increase Aβ42 accumulation and hyperphosphorylated tau compared to uninfected neurons (Powell-Doherty et al., 2020). Taken together, findings from neuronal culture, animal models and human autopsy studies implicate the hippocampus as the nexus between HSV-1 and memory-related disorders, such as AD and aMCI/MCI.

## Susceptibility Factors Toward HSV-1 Infection in the Hippocampus

Several biological factors potentially place the hippocampus at risk for HSV-1 infection compared to other brain regions, providing a mechanistic basis for the hippocampal tropism of HSV-1 (Table 2). For one, receptors for HSV-1 cellular entry are highly expressed in the hippocampus. The hippocampus is a site of active neurogenesis throughout adulthood, which may also favor HSV-1 infection. The impaired antiviral immunity in the hippocampus, especially during aging, may further render the hippocampus vulnerable to HSV-1 infection. HSV-1 may also capitalize on the high levels of hippocampal glucocorticoid receptors (GRs) to promote its virulence. Additionally, the high APP levels in the hippocampus may facilitate HSV-1 neuronal propagation. Details of these hippocampal susceptibility factors are further discussed below.

**TABLE 2:** Susceptibility factors of the hippocampus toward HSV-1 infection.

Susceptibility factor	Component	Function
High expression of viral receptors	↑ NMMHC-IIA (MYH9) ↑ MAG ↑ HVEM (TNFRSF14) ↑ Nectin-1 (PVRL1 or HveC)	Binds to gB for HSV-1 attachment Binds to gB for HSV-1 fusion and entry
Abundance of NPCs/NSCs: A neurogenic niche	↑ HSPG	Binds to gB for HSV-1 attachment
Inadequate antiviral immunity	↓ IL-6 ↓ Microglial type I interferon	Lowered resistance against HSV-1 infection
High expression of GR	↑ GR	Interact with HSV-1 promoters to enhance infectivity
High expression of APP	↑ APP	Promote HSV-1 spread

*Alternative names are bracketed; refer to the main text for relevant references. APP, amyloid precursor protein; GR, glucocorticoid receptor; HSPG, heparan sulfate proteoglycan; HveC; herpesvirus entry mediator C; HVEM, herpesvirus entry mediator; IL-6, Interleukin-6; MAG, myelin-associated glycoprotein; MHY9, myosin heavy chain 9; NMMHC-IIA, non-muscle myosin heavy chain-IIA; PVRL1, poliovirus receptor-like 1; TNFRSF14, tumor necrosis factor receptor superfamily, member 14.*

### High Expression of Cellular Receptors for HSV-1

HSV-1 entry and infection in cells rely on the presence of viral envelop gB, gD and gH/gL and cell surface receptors for gB and gD. According to the Allen Brain Atlas transcriptome database of the adult human brain, the expression of receptors for the envelop glycoproteins of HSV-1, specifically gB (i.e., NHMC-IIA and MAG receptors) and gD (i.e., HVEM and nectin-1 receptors), were found to be highest in the hippocampus by 2–3-fold compared to other brain regions (Lathe and Haas, 2017). The same study also found similar HSV-1 receptors being highly expressed in the murine hippocampus (Lathe and Haas, 2017). Immunohistochemical analyses have also revealed that nectin-1 expression was particularly high in the hippocampus of mice and humans (Horvath et al., 2006; Prandovszky et al., 2008). Similarly, nectin-1 RNA was detected in large quantities in the murine hippocampus compared to other brain regions (Haarr et al., 2001). Aside from nectin-1, the distribution of other HSV-1 receptors in the brain has not been widely studied.

Furthermore, it was shown that the cerebellum lacks gD receptors (Lathe and Haas, 2017), which may explain the finding that HSV-1 inoculation into the cerebellum did not induce lethal disease in mice (McFarland and Hotchin, 1987). This was in contrast to the pervasive viral spread and death when HSV-1 was inoculated into the murine hippocampus instead (McFarland and Hotchin, 1987). Another study also showed that HSV-1 binds more strongly to the murine hippocampus than the brainstem and cerebellum (McFarland et al., 1982). Based on animal models investigating HSV-1 spread in the brain, HSV-1 infects the hippocampus in most studies, and rarely targets the cerebellum (Table 1). Therefore, the HSV-1 tropism for the hippocampus may be attributed to the high expression of viral gB and gD receptors in the hippocampus.

### Abundance of NSCs/NPCs: A Neurogenic Niche

As demonstrated *ex vivo*, the hippocampus and periventricular areas of neonate mice were particularly susceptible to HSV-1 infection (Braun et al., 2006). The viral dissemination into these brain regions where neuronal differentiation is active suggests that dividing cells are more vulnerable to HSV-1 infection (Braun et al., 2006). Using organotypic hippocampal cultures, it was shown that the hippocampal DG (i.e., the chief neurogenic niche) was most vulnerable to HSV-1 infection compared to hippocampal glia and other neuronal types (Ando et al., 2008). Another study also showed that HSV-1 preferentially infects undifferentiated NSCs rather than mature hippocampal neurons, resulting in impaired hippocampal neurogenesis (Li Puma et al., 2019). More recent studies have shown that HSV-1 readily infects NSCs/NPCs and induces Aβ42 accumulation, neuroinflammation and neuronal impairments, which can be prevented with valacyclovir antiherpetic treatment (Abrahamson et al., 2020; Cairns et al., 2020; Zheng et al., 2020).

The vulnerability of dividing, undifferentiated NSCs in the hippocampal DG to HSV-1 infection could be attributed to the high expression of surface HSPGs. HSPGs comprise a family of two glycoproteins, syndecans and glypicans, which are highly expressed throughout mammalian neurogenesis (Hagihara et al., 2000; Wang et al., 2012; Oikari et al., 2016; Yu et al., 2017). HSPGs regulate basic fibroblast growth factor (bFGF; NSCs mitogen) to initiate neurogenesis (Rapraeger et al., 1991; Yayon et al., 1991; Vicario-Abejon et al., 1995). However, HSPGs also mediate HSV-1 attachment to mammalian cell surfaces (WuDunn and Spear, 1989; Herold et al., 1991, 1994). HSV-1 infection in mice has also been shown to downregulate FGF-2 expression and NSCs proliferation (Rotschafer et al., 2013). Similarly, reactivating HSV-1 in mice resulted in Aβ40/42 accumulation in the hippocampal NSCs, disrupting neurogenesis (Li Puma et al., 2019). Therefore, HSPGs play dual roles in promoting NSCs proliferation and HSV-1 cell attachment.

In addition, surface HSPGs have been implicated in the pathogenesis of AD (Zhang et al., 2014). HSPGs expression has been detected in Aβ plaques and NFTs in cortical areas and more frequently in the hippocampus of AD patients (Snow et al., 1992; Bignami et al., 1994; Verbeek et al., 1999). This indicates that existing NFTs and Aβ plaques in the hippocampus may bind to HSV-1 via HSPGs, perhaps to advance AD progression. Indeed, the heparin-binding domain of Aβ oligomers has been shown to bind to HSV-1 glycoproteins, which entrapped and neutralized HSV-1 to prevent encephalitis in mice, but at the consequence of increased Aβ42 accumulation (Eimer et al., 2018). Conversely, HSV-1 infection has been shown to form a protein corona layer that bound to amyloidogenic peptides and catalyzed Aβ42 accumulation in the hippocampus and cortex of mice (Ezzat et al., 2019). Taken together, HSPGs-mediated interactions between HSV-1 and Aβ peptides at the NSCs-rich hippocampus may initiate and/or facilitate AD neurodegenerative processes.

### Inadequate Antiviral Immunity

NPCs have also been found to be susceptible to HSV-1 infection and latency establishment in murine and neuronal 3D models (Menendez et al., 2016; Zheng et al., 2020). In cultured NPCs, HSV-1 infection decreased neuronal survival, which was prevented in co-cultures of NPCs with microglia (Chucair-Elliott et al., 2014). This protective effect can be reversed by the addition of IL-6-specific neutralizing antibodies. Likewise, exposing NPCs to recombinant IL-6 demonstrated similar protective effects against HSV-1 infection (Chucair-Elliott et al., 2014). IL-6 activation has also been associated with increased *in vivo* resistance against HSV-1 infection (Carr and Campbell, 1999; LeBlanc et al., 1999a). Immunohistochemical analyses have revealed that IL-6 expression was localized in the ventricles and lesser in other areas, including the hippocampus, in mice (Aniszewska et al., 2015). Hence, the low protein levels of IL-6 in the hippocampus may not provide sufficient antiviral immunity against HSV-1 infection.

Microglia have been identified as the primary activator of cyclic GMP-AMP synthase-stimulator of interferon genes (cGAS-STING)-dependent type I interferon antiviral defense against HSV-1. Specifically, mice deficient in cGAS or STING showed impaired microglial type I interferon responses and elevated HSV-1 replication in the brain, leading to increased vulnerability to HSE (Reinert et al., 2016). A genome-wide study analyzing the microglia immunophenotype in the adult mice brain found that the immune vigilance (e.g., antiviral interferon activities) of hippocampal microglia was more robust than other brain areas. Interestingly, the hippocampal microglia were most vulnerable to age-related decline in immune function (Grabert et al., 2016). HSV-1 might become opportunistic as a result, targeting the hippocampus when microglial immunosurveillance weakens. This is consistent with the findings that senescence or aged microglia often preceded AD-related neuropathology in the brain, including the hippocampus (Kaneshwaran et al., 2019; Rodriguez-Callejas et al., 2020), as also reviewed in Streit et al. (2009). Henceforth, specific antiviral immunity against HSV-1 might be inadequate in brain regions susceptible to HSV-1 infection. Kramer and Enquist (2013) also hypothesized that not all cells of the nervous system are equally prone to HSV-1 infection due to variations of immune defenses involved.

### High Expression of Glucocorticoid Receptor (GR)

In the mammalian brain, high GR expression has been found in the hippocampus throughout life (Reul et al., 1989; Wang et al., 2013). Hence, the hippocampus is known to be highly vulnerable to glucocorticoid- or stress-related pathology, as reviewed in McEwen et al. (2016). Activated GR is known to interact with viral promoters to facilitate viral replication and infectivity in the brain (Fouty and Solodushko, 2011). The HSV-1 genome has several GR response elements that have been shown to stimulate viral promoters (i.e., VP16 and ICP0) to initiate reactivation and replication (Harrison et al., 2019; Ostler et al., 2019). These studies further demonstrated that GR antagonists prevented HSV-1 shedding in neuronal cells and reactivation in mice (Harrison et al., 2019; Ostler et al., 2019). Inhibiting glucocorticoid synthesis with cyanoketone also inhibited HSV-1 reactivation in mice (Noisakran et al., 1998). In contrast, dexamethasone (i.e., synthetic glucocorticoid) treatment has been shown to induce HSV-1 reactivation and replication *in vitro* and *in vivo* (Sawiris et al., 1994; Halford et al., 1996; Hardwicke and Schaffer, 1997; Noisakran et al., 1998; Erlandsson et al., 2002; Du et al., 2012; Harrison et al., 2019). Therefore, the hippocampus has a prominent GR expression that could promote HSV-1 virulence in the CNS.

### High Expression of Amyloid Precursor Protein (APP)

HSV-1 infection has been shown to upregulate enzymes that cleave APP following the amylogenic pathway to generate Aβ40/42 peptides (Wozniak et al., 2007; De Chiara et al., 2010; Piacentini et al., 2015). APP is ubiquitously expressed in the brain, with higher expression found in the olfactory system, cerebral cortex and hippocampus (Card et al., 1988; Imaizumi et al., 1993). These areas are also known to be targeted by HSV-1 (Table 1). HSV-1 capsids have been shown to bind to APP to expedite viral transport in both squid and epithelial cell culture models (Satpute-Krishnan et al., 2003; Cheng et al., 2011). The infected epithelial cells further displayed abnormal APP processing, resulting in mislocalized APP that may contribute to AD (Cheng et al., 2011).

Recent studies have also demonstrated that HSV-1 infection induced Aβ42 or Aβ40/42 accumulation, indicative of pathological APP metabolism, in the hippocampus *in vitro* and *in vivo* (De Chiara et al., 2019; Ezzat et al., 2019; Powell-Doherty et al., 2020). Interestingly, Aβ40/42 accumulation was observed in the hippocampus in a mouse model of HSV-1 reactivation, but such neuropathology did not occur in mice with APP gene knockout (Li Puma et al., 2019). Thus, APP appears imperative for the cellular propagation and spread of HSV-1, generating Aβ40/42 peptides in the process. This might also contribute to the hippocampal susceptibility to HSV-1 infection, given that the hippocampus has high APP expression.

## Concluding Remarks

The review discussed the interplay between HSV-1 and hippocampal- or memory-related brain disorders, namely AD and aMCI/MCI. Next, this review outlined the theoretical pathway by which HSV-1 productive infection or reactivation infiltrates the brain, underscoring its predilection for the limbic system and the hippocampus therein. HSV-1 likely induces neuropathological effects in the hippocampus comparable to AD phenotype. Given the established role of the hippocampus in learning and memory, aMCI/MCI likely precede AD in the course of disease development in persistent or recurrent HSV-1 infection.

Factors and mechanisms contributing to the hippocampal susceptibility to HSV-1 infection are also elucidated. Several 2D and 3D cell culture studies reported the use of antiherpetic agents to prevent HSV-1-induced AD-related neuropathology, including hippocampal damage (Ando et al., 2008; Wozniak et al., 2011, 2013; Cairns et al., 2020). This is consistent with three large retrospective cohort studies spanning multiple countries showing that antiherpetic agents (e.g., acyclovir and valacyclovir) were associated with a reduced risk of dementia (Tzeng et al., 2018; Lopatko Lindman et al., 2021; Schnier et al., 2021). However, observational cohort studies can only inform associations, not causation. To this end, an on-going 78-week phase II randomized placebo-controlled clinical trial is assessing the efficacy of valacyclovir in attenuating symptom progression in patients with mild AD with HSV-1 seropositivity (Devanand et al., 2020). This is the first trial to investigate whether antiviral has any causal role in treating AD (Devanand et al., 2020).

It is still unknown whether the risk of AD development or progression would remain attenuated should antiviral agents be discontinued as HSV-1 may reactivate thereafter. Current antiherpetic agents only inhibit HSV-1 replication and do not eradicate HSV-1 latency (LeBlanc et al., 1999b; Sawtell et al., 2001). Hence, HSV-1 may reside permanently in the nervous system amongst those infected, with their hippocampal function at risk for HSV-1 infection. More research could be conducted on potential treatments that may attenuate or prevent HSV-1-induced neuropathology. For one, the optimal drug dosage, frequency and duration of antiherpetic agents in treating AD should be determined, in light of HSV-1 latency. No vaccines are available for HSV-1 to date, suggesting further research on vaccine design and development to be considered (Whitley and Baines, 2018). Multiple phase II/III clinical trials investigating Aβ-based therapies (e.g., secretase inhibitors and monoclonal antibodies) for AD have been unsuccessful, as reviewed in Oxford et al. (2020). A possible reason for this could be an on-going HSV-1 infection or reactivation that may promote the formation or prevent the clearance of Aβ40/42 in the brain, especially in the hippocampus. Therefore, synergistic antiherpetic agent with Aβ-based therapy may show promise in treating AD.

**FIGURE 2:**
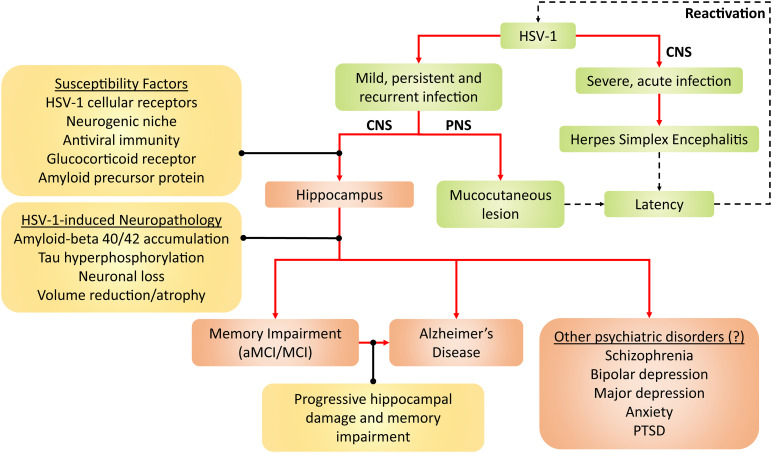
Framework for HSV-1 pathogenicity. Green denotes established disease pathways wherein mild HSV-1 infection causes mucocutaneous lesions of the lips (cold sores), genitals (genital herpes), and cornea (keratitis). Severe HSV-1 infection causes herpes simplex encephalitis (HSE). Following productive infection (dashed arrows), HSV-1 establishes latency in the trigeminal ganglion and olfactory bulb, and periodically reactivates. Red-brown denotes current and emerging putative pathogenicity pathways, wherein HSV-1 preferentially infects the hippocampus due to several susceptibility factors. HSV-1 consequently induces neuropathological effects and, thus, compromises hippocampal functions. As a result, memory becomes impaired, which may lead to aMCI/MCI and AD. Alternatively, the progressive HSV-1-induced hippocampal damage may facilitate the progression from aMCI/MCI to AD. Given that hippocampal dysfunction may be present in other neuropsychiatric disorders such as schizophrenia, PTSD, depressive and anxiety disorders, HSV-1 may hypothetically contribute to such disorders as well. Aβ, amyloid-beta; AD, Alzheimer’s disease; aMCI, amnestic mild cognitive impairment; CNS, central nervous system; HSV-1, herpes simplex virus type 1; MCI, mild cognitive impairment; PNS, peripheral nervous system; PTSD, post-traumatic stress disorder.

All in all, persistent HSV-1 infection and reactivation may present as risk factors, which likely interacts with and adds to other risk factors (e.g., age, ApoE4 genotype and other microbial infections) in the development of AD and other hippocampal-related brain disorders, as reviewed in Wainberg et al. (2021) and Vigasova et al. (2021). However, there is no evidence of causation in humans yet as HSV-1 reactivation cannot be measured in the living brain, as Mancuso et al. (2019) suggested. Although only AD and aMCI/MCI neuropathogenesis have been strongly linked to HSV-1, HSV-1 may also be involved in other hippocampal-related brain disorders, such as schizophrenia, depression, anxiety and post-traumatic stress disorder (Small et al., 2011; Anand and Dhikav, 2012; Klein, 2017). This possibly adds on to the list of established diseases caused by HSV-1, namely mucocutaneous lesions and encephalitis (Figure 2).

## Author Contributions

SY wrote the manuscript. SY, JC, and WL conceptualizedand edited the manuscript. MY, ST, TS, and IP critically revised the manuscript. All authors read and approved the manuscript.

## Conflict of Interest

The authors declare that the research was conducted in the absence of any commercial or financial relationships that could be construed as a potential conflict of interest.

## Publisher’s Note

All claims expressed in this article are solely those of the authors and do not necessarily represent those of their affiliated organizations, or those of the publisher, the editors and the reviewers. Any product that may be evaluated in this article, or claim that may be made by its manufacturer, is not guaranteed or endorsed by the publisher.
